# On the Treatment of Suspended Animation under the Influence of Chloroform

**Published:** 1862-08

**Authors:** William Marcet

**Affiliations:** Assistant-Physician to the Westminster Hospital, etc., etc.


### ON [illegible]HE TREATMENT OF SUSPENDED ANIMATION [illegible]NDER THE INFLUENCE OF CHLOROFORM.

---

By [illegible]ILLIAM MARCET, M.D., F.R.S., Assistant-Physician to the Westminster Hospital, etc., etc.

---

In the *Medical Times and Gazette*, for July 20, I offered a few remarks on the phenomena attending the accumulation of vapors of c[illegible]loroform in the blood, and insisted on the importance of watching [illegible]he state of the respiration as well as that of the pulse during [illegible]he exhibition of this anæsthetic agent. The number for Octob[illegible] 28, of the same periodical, containing two new cases of death [illegible]rom chloroform, I may be perhaps allowed to return to this s[illegible]ject, my present object being to suggest a mode of treatment in [illegible]ese cases which to my knowledge has not yet been proposed; [illegible]d considering the failure which has nearly constantly attend[illegible] every attempt to restore animation suspended by an overdos[illegible]r under the influence of chloroform, the following suggestions [illegible]ill be most probably of interest, and also, I trust, of practical [illegible]ility.

It has frequently [illegible]curred to me that, in many instances, the final cause of death [illegible]m chloroform was owing, not only to its anæsthetic properties[illegible]ut also partly to spasm of the glottis. I do not mean, howeve[illegible] that the passage of the vapor of chloroform through the glottis and larynx has the power of causing an involuntary closure of the glottis; and I cannot agree with Dr. Black, of St. Bartholomew's Hospital, who states:—"Any concentration of the vapor of chloroform which can be breathed is safe; any condition of dilution which causes the patient to cough or to hold his breath is dangerous, and if persevered in for half a minute, may be fatal." I believe a spasmodic closure of the glottis to take place occasionally, from the action of the chloroform which has been absorbed into the blood, and that this obstacle to the admission of air into the lungs, taken in connection with the narcotic or anæsthetic effect of the chloroform circulating in the blood, has been in some, perhaps many, cases the actual cause of death. Should this view be correct, it will follow that if air be allowed to enter the lungs by means of tracheotomy, or by opening the glottis with a trache-tube, or any other operation which will effect the same purpose, life will in such cases be saved.

It may be inferred that spasm of the glottis takes place occasionally under the influence of chloroform.

1st. Because several substances possessing anæsthetic properties are positively known, when present in the blood, to have given rise to closure of the glottis.

2nd. Because the symptoms of death from chloroform are consistent, more or less, with death from sudden asphyxia.

3rd. Because the *post mortem* appearances after death from chloroform may be accounted for by assuming that death has taken place from asphyxia.

In the three following cases, spasmodic closure of the glottis resulted from the presence of alcohol, carconic acid, and sulphuretted hydrogen in the blood, the physiological properties of these three substances being allied, to a certain extent to those of chloroform; and in two of the instances under our consideration, life was obviously saved by tracheotomy.

A very interesting case is reported in the volume of the Medico-Chirurgical *Transactions* for 1837, entitled, "Case of Recovery from the Insensibility of Intoxication by the Performance of Tracheotomy. By George Samson, Esq." The patient, aged 31, was brought to Mr. Samson's house in a state of complete insensibility, after drinking freely of beer, and more than a pint of brandy; all voluntary motions had ceased for at least four hours. The stomach-pump being used, drew off between three and four pints of fluid, the greater part of which appeared to consist of brandy. Every means of exciting vomiting was afterwards vainly applied; the man became more comatose, his countenance turgid, and breathing more and more difficult; the pulse grew fainter, and was at last scarcely perceptible. He was then removed to the Infirmary, and a consultation was held with other medical attendants, who arrived in the course of half an hour ; at that time every appearance indicated the rapid approach of death, and there was no ground to justify a reasonable hope of recovery. It occurred to Mr. Sampson, when standing by the patient's bed-side, that the extreme difficulty of respiration was owing to the existance of "collapse of the glottis," and with this view of the case, he strongly urged that a trial should be given to the operation of tracheotomy. The operation was accordingly performed, without loss of time, by Mr. Andrews. The trachea was no sooner opened, than the distension of the veins about the head and neck subsided, the violent efforts of the respiratory muscles ceased, and in about half an hour regular and easy respiration through the wound was freely established. At the same time the pupils became slightly sensible to the stimulus of light, and the pulse returned to the wrist. He continued quiet during the night, but had no return of consciousness till the following morning. The case proceeded very satisfactorily, and the wound being healed in about three weeks, the patient was discharged cured.

This case is particulary interesting, for the analogy between the physiological action of alcohol and chloroform has been quite satisfactory demonstrated by Messrs. Lallemand, Perrin, and Duroy. Like alcohol, chloroform acts first on the brain, then on the spinal system, and finally on the sympathetic; the brain exerts a certain power of concentrating within its tissue both chloroform and alcohol; the period of excitement produced by chloroform, is not unlike that of alcoholic intoxication; and insensibility equally results when a sufficient dose of alcohol or chloroform has penetrated into the circulation.

If alcoholic poisoing is positively shown to have threatening life from asphyxia owing to spasm of the glottis, I see no reason why death from chloroform should not be due occasionally to the same phenomenon.

The two other cases I have to report have come under my own observation. One of them was an instance of secondary asphyxia, from spasm of the glottis, after immersion in the Serpentine during the skating season. The patient, a middle-aged man, had been entirely under water, but on being taken out, respiration returned, and continued comparatively free until placed in a warn bath, when he suddenly exhibited alarming signs of asphyxia, obviously from spasmodic closure of the glottis. A few minutes later the patient was removed to a bed, when several similar attacks occurred, one of them still more severe than the first. He recovered in the course of some hours.

This is an instance of spasm of the glotts produced by an accumulation of carbonic acid in the circulation. The gas in question possesses anæsthetic properties, and is so far allied to chloroform.

The third case is one of equal interest, although the subject of the observation was a dog. About two years ago, when engaged in injecting an aqueous solution of sulphuretted hydrogen into the external jugular vein of a dog, I observed that the animal's respiration, instead of becoming somewhat deeper, as usually happens during this operation, began to fail, and shortly afterwards ceased, without their being the slightest struggle or apparent symptom of asphyxia from the closure of the gottis. I immediately prepared to have recourse to artificial respiration by means of an instrument I have invented for that purpose. In order to insert a tube into the trachea, I incised this organ, when immediately, to my surprise, the animal commenced breathing through the opening. After a few minutes, free respiration and sensibility returned, and the animal recovered perfectly. It is obvious that, in the present instance, the respiraton had been arrested by closure of the glottis, and the animal was dying from asphyxia; had not tracheotomy been performed, the dog would have died with every symptom of syncope. This case, which I report from memory, was witnessed by many of the pupils of the Westminster Hospital. It shows two interesting facts.

1st. That the presence of an excessive quantity of sulphuretted hydrogen in the blood may cause death from spasm of the glottis; and, 2nd. That on those occasions death takes place without the struggles or convulsions so peculiar to impeded respiration. Consequently, in cases of death from chloroform, the absence of convulsions is no proof that there exists no mechanical obstical to the free admission of air into the lungs.

The symptoms of death by chloroform are consistent with those of asphyxia. Dr. Snow's book contains a very interesting case of death from chloroform, with symptoms of asphyxia, which was communicated by Dr. Solly to the *Medical Gazette.* This case (No 12) bears directly on the subject of the present communication. The patient, a porter, aged 48, and apparently in perfect general health, was submitted to chloroform for the removal of a toe-nail. The anæsthetic vapors were administered by means of an inhaler; after the operation had been performed, and being still insensible, the patient's face became dark, his pulse small, quick, but regular, respiration laborious; his neckerchief was removed and chest exposed to fresh air from a window near to the bed, cold water was dashed on his face, the chest rubbed, and ammonia applied to the nose. After struggling for about a minute, he became still, the skin cold, pulse scarcely perceptible, and soon ceased to be felt at the wrist. Immediately on the appearance of these symptoms artificial respiration was commenced by depressing the ribs with the hands and allowing them to rise again, until the proper apparatus was brought, when respiration was kept up by means of the trachea-tube and bellows, and oxygen gas introduced into the lungs by the same means. Galvanism was also applied, but to no purpose.

Dr. Snow considers there is some obscurity about the above narrative, which I have given as nearly in the same words as those used in the report; in his opinion the symptoms exhibited would be inconsistent with death from chloroform. It appears to me, however, that this is clearly a case of asphyxia from spasm of the glottis under the influence of chloroform, and I cannot help believing that had treacheotomy been performed at the time when artificial respiration was commenced, the patient would have been saved. If most cases of death from chloroform are not attended with evident signs of asphyxia, still this is no argument in favor of the absence of spasm of the glottis, for it must be remembered that poisoning by chloroform is a complex phenomenon which results from excessive anæsthesia producing a tendency to paralysis of the muscles, and an action on the heart predisposing to death by cardiac syncope; any epasmodic closure of the glottis occurring under these circumstances would, it may be anticipated, cause sudden death without the recurrence of convulsions, or struggles for breath.

The *post mortem* appearances in cases of death from chloroform do not preclude the idea of death from asphyxia; indeed in cardiac syncope, which is according to Dr. Snow the fatal termination of poisoning by chloroform,—"If the blood have not been displaced by artificial respiration or other causes, the right cavities of the heart and the adjoining great veins will be found filled with blood, and the lungs will in many case be more or less congested. The appearances, in short, will be very much the same as in asphyxia by privation of air which ends in a kind of cardiac syncope." Nothing can be more in favor of the view I am advocating. It must be well understood that I do not consider every case of fatal poisoning by chloroform as owing to spasm of the glottis, and there is every reason to believe that in some instances death takes place from paralysis of the muscles of respiration, in others from cardiac syncope, or from a simultaneous occurrence of both these effects.

If it be admitted that death from chloroform be occasionally owing to spasm of the glottis, then the importance of performing tracheotomy in these cases, and adopting some means of allowing air to enter freely through the wound, will be readily understood. It must be remembered that the cases on record of recovery from suspended animation owing to an overdose of chloroform are very few, and, as a rule, it may be considered that, after the respiration has ceased, and the pulse become hardly perceptible at the wrist, death is inevitable. Under these circumstances, any means apparently available should be adopted. The operation of tracheotomy ought to be performed as soon as possible after the respiration has stopped, and the patient assumed that livid countenance known in these cases to precede death; the loss of every second diminish the chance of saving life. Artificial respiration, if possible, must however not be neglected, and should be carried on before, during, and, if necessary, after the operation of tracheotomy has been performed. Artificial respiration is of great importance in connexion with death from chloroform, and I am at present busily engaged inquiring into this subject.—*Medical Times and Gazette, and Boston Journal.*

